# Factors Determining Immunological Response to Vaccination against Tick-Borne Encephalitis Virus in Older Individuals

**DOI:** 10.1371/journal.pone.0100860

**Published:** 2014-06-26

**Authors:** Pontus Lindblom, Peter Wilhelmsson, Linda Fryland, Andreas Matussek, Mats Haglund, Johanna Sjöwall, Sirkka Vene, Dag Nyman, Pia Forsberg, Per-Eric Lindgren

**Affiliations:** 1 Division of Medical Microbiology, Department of Clinical and Experimental Medicine, Linköping University, Linköping, Sweden; 2 Division of Infectious Diseases, Department of Clinical and Experimental Medicine, Linköping University, Linköping, Sweden; 3 Division of Medical Services, Department of Microbiology, County Hospital Ryhov, Jönköping, Sweden; 4 Department of Infectious Diseases, County Hospital Kalmar, Kalmar, Sweden; 5 Clinic of Infectious Diseases, Linköping University Hospital, Linköping, Sweden; 6 Public Health Agency of Sweden, Stockholm, Sweden; 7 Åland Central Hospital, Mariehamn, Åland, Finland; University of Minnesota, United States of America

## Abstract

We performed a cross-sectional study including 533 individuals (median age 61) from the highly TBE endemic Åland Islands in the archipelago between Sweden and Finland. Blood samples, questionnaires and vaccination records were obtained from all study participants. The aim was to investigate if there was any association between TBEV antibody titer and 12 health-related factors. Measurement of TBEV IgG antibodies was performed using two commercial ELISA assays (Enzygnost and Immunozym), and a third in-house rapid fluorescent focus inhibition test was used to measure TBEV neutralizing antibodies. The age of the individual and the number of vaccine doses were the two most important factors determining the immunological response to vaccination. The response to each vaccine dose declined linearly with increased age. A 35 year age difference corresponds to a vaccine dose increment from 3 to 4 to achieve the same immunological response. Participants previously vaccinated against other flaviviruses had lower odds of being seropositive for neutralizing TBEV antibodies on average, while participants with self-reported asthma had higher odds of being seropositive. By comparing the 3 serological assays we show that the Enzygnost and Immunozym assay differ due to choice of cutoffs, but not in overall accuracy.

## Introduction

Tick-borne encephalitis virus (TBEV) is responsible for one of the most serious viral neuroinfections in Europe and Asia, manifesting as meningitis, encephalitis or meningoencephalitis, which can lead to death or long term morbidity [1]–[3]. TBEV is a member of the genus Flavivirus, which also includes the mosquito-borne viruses; yellow fever, Japanese encephalitis, dengue and West Nile [4]. Flaviviruses are spherical enveloped particles, 40–60 nm in diameter, with 10–11 kb long ssRNA(+) genomes. TBEV is transmitted through the bite of an infected tick of the species *Ixodes ricinus* (European TBEV subtype) or *I. persulcatus* (Far Eastern and Siberian TBEV subtypes) [5]. The virus is endemic on the Eurasian continent from the Balkan Peninsula in the south-east to Scandinavia in the north, and from eastern France in the west throughout central Eurasia to the Japanese Islands in the east [6]. During the last 20 years, 5,000–13,000 human clinical cases of tick-borne encephalitis (TBE) have been reported annually, with the majority from Russia [6]. The incidence of TBE is highest among older individuals [7], in whom the disease is also more severe [1]–[3], and more men than women contract TBE in Europe [8], [9]. There is currently no specific antiviral treatment available [10]. Prevention of TBE relies on vaccination and measures to prevent tick-bites. Two vaccines are available in Europe for active immunization against TBEV; FSME-IMMUN (Baxter, Austria), and Encepur (Novartis, Germany). Both vaccines are considered safe and efficacious for individuals ≥1 year [11]. In Russia and a few neighboring countries, two vaccines based on Far Eastern TBEV strains are available; TBE Moscow Vaccine (Chumakov Institute, Russia), and EnceVir (Microgen, Russia) [12]. Studies suggest that all four vaccines give cross-protection against all 3 subtypes of TBEV [12]–[14].

Mass vaccination of a population can reduce the total number of TBE cases by up to 90% as demonstrated in Austria in the 1980s [15]. By comparing TBE incidence between the vaccinated and unvaccinated population in Austria 2000–2011, the field effectiveness for regularly vaccinated individuals has been calculated to be 96–99%, and estimated to have prevented >4,000 cases of TBE in the country during that time period [7]. The World Health Organization (WHO) recently published a position paper on TBE [11], recommending vaccination for whole populations in highly endemic areas (>5 cases/100,000/year), and vaccination of risk groups in low to moderate endemic areas (<5 cases/100,000/year).

A primary vaccination requires 3 doses the first year (months 0, 1, 5–12). After 3 years a booster dose is needed, and subsequent boosters at intervals of 5 years, or 3 years if ≥60 years [16]. The duration of protection after vaccination has only been studied indirectly by measuring titers of antibodies against TBEV as a surrogate marker of protection.

Several studies have shown that the antibody response to TBEV vaccination declines with age, resulting in a significantly higher proportion of individuals over 50 years of age being seronegative 2–10 years after the last vaccine dose [17]–[23]. The antibody response to TBEV vaccination appears to decline linearly throughout adult life [17]. Although age affects the quantitative antibody response, the quality of the antibodies appears unaffected by increased age [24]. In contrast to vaccination, individuals naturally infected with TBEV preserve high antibody titers throughout life [22]. Vaccine failures can occur in all ages, despite complete vaccination, but the majority of cases are among individuals over 50 years old [15], [25], [26]. Most vaccine failures are characterized by a delayed IgM antibody response, and high titers of neutralizing IgG antibodies present in the first samples taken upon hospitalization [27].

Antibody titers are considered to correlate with protection. Therefore, it is important to know the performance of the methods used for measuring antibody titers. The Baxter vaccine, FSME-IMMUN, is based on the Neudörfl TBEV strain, as is the Immunozym ELISA assay. The Novartis Encepur vaccine on the other hand, uses the K23 TBEV strain, as does the Enzygnost ELISA assay. A study by Jílková et al. [28] found that the results differed significantly between the two ELISA assays depending on whether the TBEV strain was heterologous or homologous with respect to the vaccine used. Neutralizing antibody tests do not suffer from this potential bias and are considered to be the best surrogate marker of protection against TBEV [27], [29].

The Åland Islands (28,600 inhabitants) located in the archipelago between Sweden and Finland, are highly TBE endemic. The first cases of meningoencephalitis were described at the islands in the 1940:s, and the TBEV strain named Kumlinge A52 was isolated in 1959 [30]. From 1959–2005, over 300 cases of TBE were serologically verified [31], and from 1990–2010 the incidence of TBE varied between 7 and 93 cases/100,000 inhabitants/year. In 2006, a mass vaccination program was started covering the population from seven years of age and older [32].

The Åland Islands are since 2008 a part of our on-going prospective epidemiological study, denoted the tick-borne diseases (TBD) STING-study [33]–[37]. In analysis of TBEV antibodies in serum samples from study participants from the Åland Islands with a history of recent tick bite, we found that a large proportion of participants states that they were vaccinated against TBEV but were in fact seronegative. To investigate why some individuals were seronegative despite vaccination, and if necessary establish better guidelines for vaccination, or more accurate methods for antibody measurements, we examined 12 health-related factors in connection to TBEV antibody titer in serum measured using 3 different assays. We found the number of vaccine doses to be most important in an age-dependent manner and we found important differences between the assays.

## Materials and Methods

### Study design

Blood samples, questionnaires and ticks were collected from 533 individuals with history of recent tick bite from the Åland Islands (Finland), between May 2008 and December 2009, as part of the TBD STING-study. The TBD STING-study is a prospective study that aims to increase the knowledge regarding the overall risk of acquiring tick-borne diseases following a tick-bite. Blood samples and questionnaires are received from the study participants, both at time of enrollment and at a 3-month follow-up. We investigate the pathogen content of the tick and the tick feeding-time, and we follow-up the study participants to see if they develop any symptoms of infection or specific antibodies during the 3-month study period. The study participants are also asked to collect any additional ticks that bite them during the study period. More information regarding the details of the study design is available in the hitherto published papers from the TBD STING-study that was started in 2008 [33]–[35], and from the pilot study that was conducted during 2007 in the province of Östergötland, Sweden [36], [37].

In this paper we present an analysis of the TBEV antibody titer in relation to 12 health-related factors in the study population from the highly TBE endemic Åland Islands, where a majority of the population are vaccinated with 3 to 5 doses against TBEV. The ticks collected from the study participants in this study have previously been analyzed for TBEV [33], and relevant data from those tests are included in this paper (regarding 3 TBEV positive ticks detached from 3 of the study participants). All the serum samples analyzed in this paper were drawn at the 3-month follow-up, except from the 3 individuals that were bitten by TBEV infected ticks, in whom both the first and 3-month serum samples were analyzed in parallel to determine if the antibody titer changed in response to the bite of a TBEV infected tick. The blood samples were centrifuged for 10 minutes at 1500×g to separate the serum from the clot within 2 h from collection, except for a few samples that were not collected on the main island that had been in room temperature approximately 24 h before centrifugation. All serum samples were kept frozen at −70°C until analysis. Three serological methods were used for measuring the serum positivity rate (SPR) and titer of TBEV specific antibodies.

### Ethics statement

Ethical approval for the TBD STING-study was granted by the Regional Ethics Committee in Linköping (M132-06), and by the local Ethics Committee of the Åland Health Care, 2008-05-23, following the principles expressed in the declaration of Helsinki. Each study participant gave their written informed consent before entering the study.

### Vaccination records, questionnaires, and medical records

We acquired vaccination records, including the date of each vaccine dose for all of the study participants from the Åland public health care service. Only the FSME-IMMUN vaccine (Baxter, Austria) has been purchased for vaccination of the Åland Islands population. Four of the factors we investigated were acquired from the vaccination records; age, gender, number of vaccine doses (0–5), and time since the last vaccine dose. From the questionnaires we acquired information regarding previous tick-borne infections, general health status, and vaccination status for TBE as well as for yellow fever and Japanese encephalitis (Appendix S1). We included 8 health-related factors from the questionnaires in the analysis that we considered might influence the immune response; previous TBE disease [22], vaccination against other flaviviruses (yellow fever or Japanese encephalitis) [38], ≥2 tick-bites during the previous 3 months [39], pet-ownership (dog or cat) [40], asthma [41], smoking [42], allergy [43], and diabetes [44]. If a study participant visited health care for a suspected tick-borne disease during the 3-month study period we also investigated their medical records.

### Enzygnost ELISA

The Enzygnost anti-TBE IgG assay (Siemens, Erlangen, Germany) was used to analyze all serum samples using a BEP 2000 Advance System (Siemens), according to the manufacturer’s instructions. The antibody concentrations (U/ml) were interpreted according to the manufacturer’s instructions (<7 negative, 7–11 borderline, >11 positive). Borderline values were considered negative in the statistical analysis of serum positivity rate (SPR).

### Immunozym ELISA

The Immunozym FSME (TBE) IgG assay (PROGEN Biotechnik GmbH, Heidelberg, Germany) was used to analyze all serum samples, according to the manufacturer’s instructions. The antibody concentrations in Vienna units per ml (VIEU/ml) were interpreted according to the manufacturer’s instructions (<63 negative, 63–126 borderline, >126 positive). Borderline values were considered negative in the statistical analysis of SPR.

### Rapid fluorescent focus inhibition test

A in-house rapid fluorescent focus inhibition test (RFFIT) for detection of TBEV specific neutralizing antibodies was performed on all serum samples, as previously described [29], [45]. Serum dilutions of 1∶5 were tested with approximately 50×FFD_50_ (50% focus forming dose) of the Swedish TBEV strain 93–783 [46]. Neutralizing titers were expressed as the reciprocal of the serum dilution that reduced the challenge virus to one FFD_50_. Titers of ≥5 were considered positive.

### Statistical methods

Demographic data were evaluated using descriptive statistics as medians with interquartile range (IQR) for ratio variables and as percentage for categorical variables. Serum antibody titers were calculated using serum positivity rate (SPR) [defined as percentage of subjects with >11 U/ml (Enzygnost), >126 VIEU/ml (Immunozym), or neutralizing antibody titer ≥5 (RFFIT)], or using geometric mean titer (GMT) [only available for Enzygnost and Immunozym]. Fisher’s exact test was used to test differences in SPR between groups. Chi-square test for trend was used to test if there was linear trend in SPR between ordered groups. The nonparametric Kruskal-Wallis test with Dunn’s post hoc test was used to test differences in GMT between groups. Binary logistic regression was used for health-related factors regressed on SPR for all three assays, and multiple linear regression was used for health-related factors regressed on antibody titer measured using the Enzygnost and Immunozym assay. Correlations between the 15 investigated factors were assessed using Pearson correlation test. The two-tailed student t-test was used to test differences of mean age and mean number of vaccine doses between groups. The nonparametric Spearman’s rho test was used to evaluate the correlation of titers measured using Enzygnost and Immunozym. To evaluate the sensitivity and specificity of the Enzygnost and Immunozym assays, receiver-operating characteristic (ROC) curve analysis was performed in reference to the RFFIT assay as the golden standard. Statistical analyses were performed using GraphPad Prism version 5.00 for Windows (GraphPad Software, San Diego, CA), and SPSS v21 (IBM SPSS Statistics, IBM Corp., Armonk, NY), *p-*values <0.05 were considered significant.

## Results

### Demography

**Figure 1:**
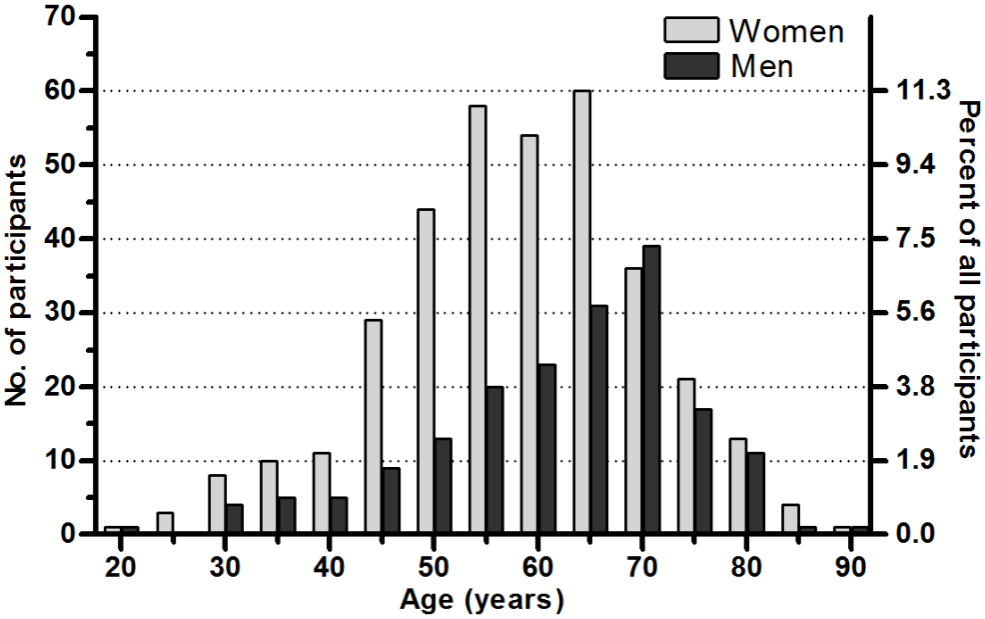
Gender and age distribution of the study participants (5-year intervals).

We analyzed serum samples received from 533 individuals from the Åland Islands from May 2008 to December 2009. For 39 of the study participants there were no records of the number of doses and dates of vaccine doses received, for this reason they were excluded from analysis relating to number of vaccine doses and time since last vaccination. Data of the study participants regarding vaccination status, and previous TBE disease are given in Table 1. Two-thirds of the participants (n = 353) were women, median age 59 (range 22–89), with 78% ≥50 years (Figure 1). For the men (n = 180), the median age was 64 (range 20–88), with 83% ≥50 years. Two study participants visited health care for a suspected tick-borne disease during the 3-month study period, both of them had erythema migrans, typical of Lyme borreliosis.

**Table 1:** Vaccination and TBE status of the study participants.

Status	Number of women	Number of men	Age, median (IQR)	Years since last vaccination, median (IQR)
0 doses	18	8	63 (50–73)	NA
1 dose	8	6	55 (44–64)	3.5 (2.3–4.5)
2 doses	7	3	62 (51–75)	1.7 (1.4–3.4)
3 doses	155	72	59 (49–68)	2.6 (1.8–2.8)
4 doses	105	50	62 (54–68)	1.4 (0.6–2.4)
5 doses	23	11	59 (52–64)	1.2 (0.5–2.0)
Previous TBE^a^	7	6	67 (62–70)	NA
0 doses+ab^b^	8	7	66 (55–75)	NA
Unknown doses^c^	22	17	57 (51–65)	NA
Total	353	180	59 (50–65)	2.4 (1.6–2.7)

IQR: Interquartile range, NA: not applicable.

a Not vaccinated according to vaccine records and the questionnaires.

b Had decided not to get vaccinated because they knew they were antibody positive, this group is distinct from the “0 doses” group.

c Stated that they were vaccinated in the questionnaire but no vaccine record on number of doses or dates of vaccination were available.

### Number of vaccine doses

**Figure 2:**
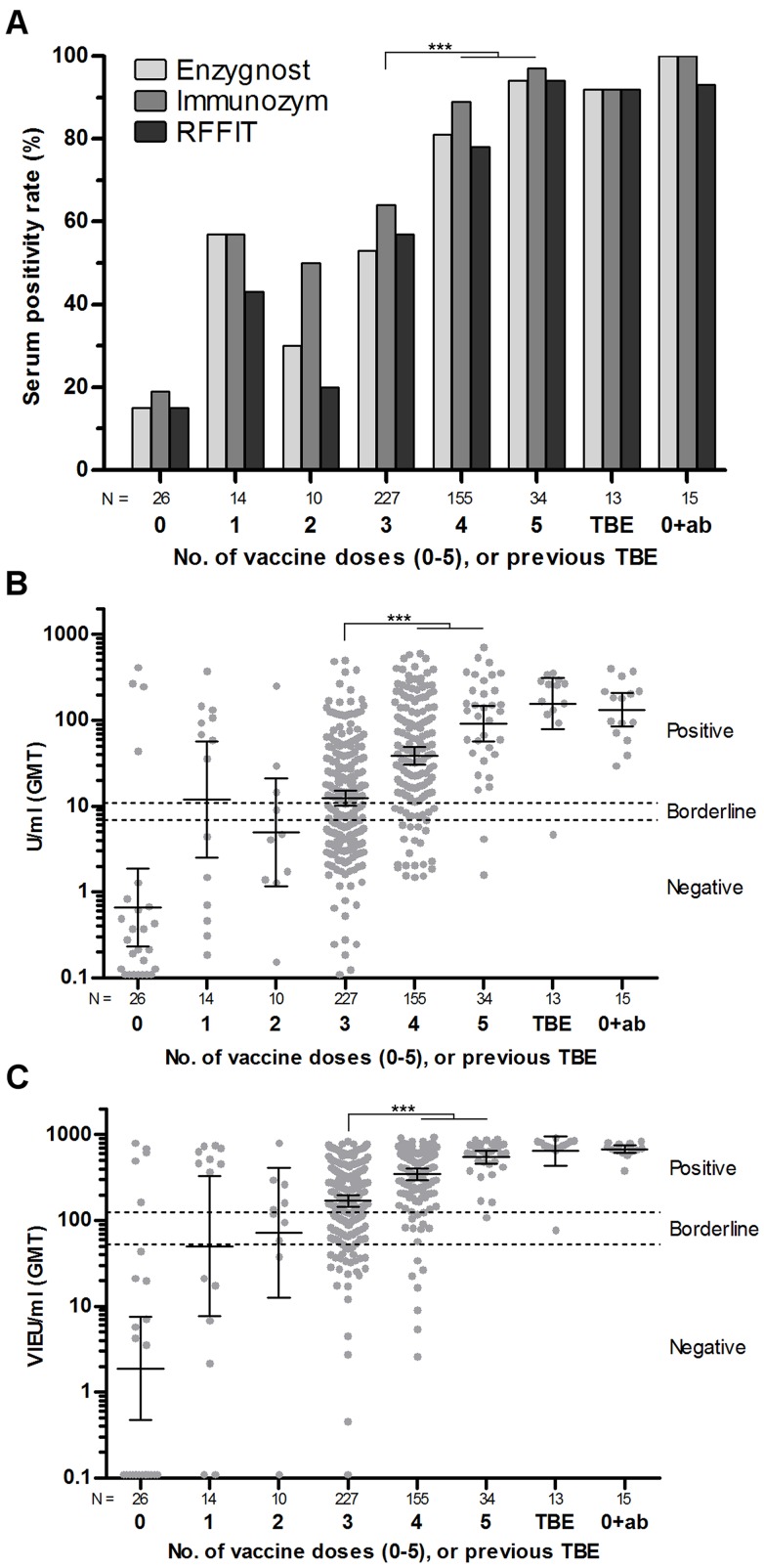
Serum positivity rate (SPR), and geometric mean titer (GMT) in relation to vaccination status. SPR and GMT in relation to number of vaccine doses (0–5), or previous TBE disease. The “0(ab)” group is individuals who decided not to get vaccinated because they knew they were antibody positive. (A) SPR for Enzygnost, Immunozym and RFFIT, (B) GMT with 95% CI measured using Enzygnost, (C) GMT with 95% CI measured using Immunozym. Both SPR (p<0.001, Fisher’s) and GMT (p<0.001, Dunn’s) was significantly higher after 4 and 5 doses compared to 3 doses, for all three assays.

The proportion of seropositive individuals with regard to number of vaccine doses were; 52% (1 dose), 33% (2 doses), 59% (3 doses), 84% (4 doses), and 96% (5 doses) (Figure 2: A). However, the dose-response after 1 and 2 doses is uncertain due to few individuals in those groups. There was a significant difference in serum positivity rate (SPR) between the groups of individuals that had received 3 vaccine doses and those that had received 4 and 5 doses (p<0.001). The difference between 3 doses versus 4 and 5 doses was also reflected in difference of geometric mean titer (GMT) (p<0.001) for both the Immunozym and Enzygnost assay (Figure 2: B, C). There was no significant difference in SPR or GMT between the groups that had received 4 or 5 doses, those who reported previous TBE disease, and those study participants that had decided not to get vaccinated because they knew that they were TBEV antibody positive.

### Age

**Figure 3:**
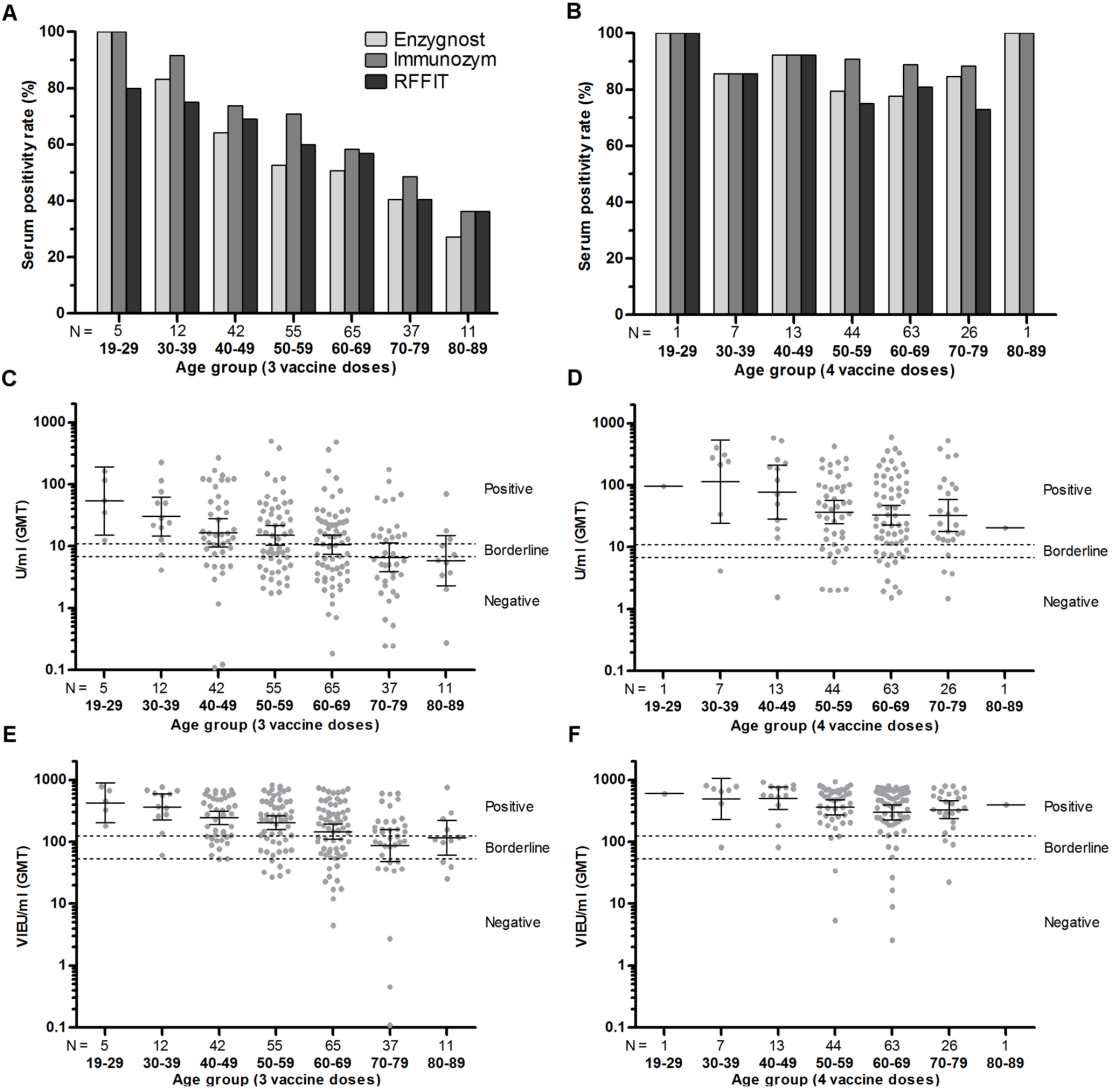
Serum positivity rate (SPR), and geometric mean titer (GMT), in relation to age group. (A, B) SPR for Enzygnost, Immunozym and RFFIT for individuals that have received 3 (A) or 4 (B) vaccine doses. (C, D) GMT with 95% CI measured using Enzygnost for individuals that have received 3 (C) or 4 (D) vaccine doses. (E, F) GMT with 95% CI measured using Immunozym for individuals that have received 3 (E) or 4 (F) vaccine doses. Both SPR (p<0.001, Chi-square test for trend) and GMT (p<0.001, Kruskal-Wallis) decreased significantly with age with all three assays for participants that had received 3 doses. The trend was not significant for participants that had received 4 doses.

**Figure 4:**
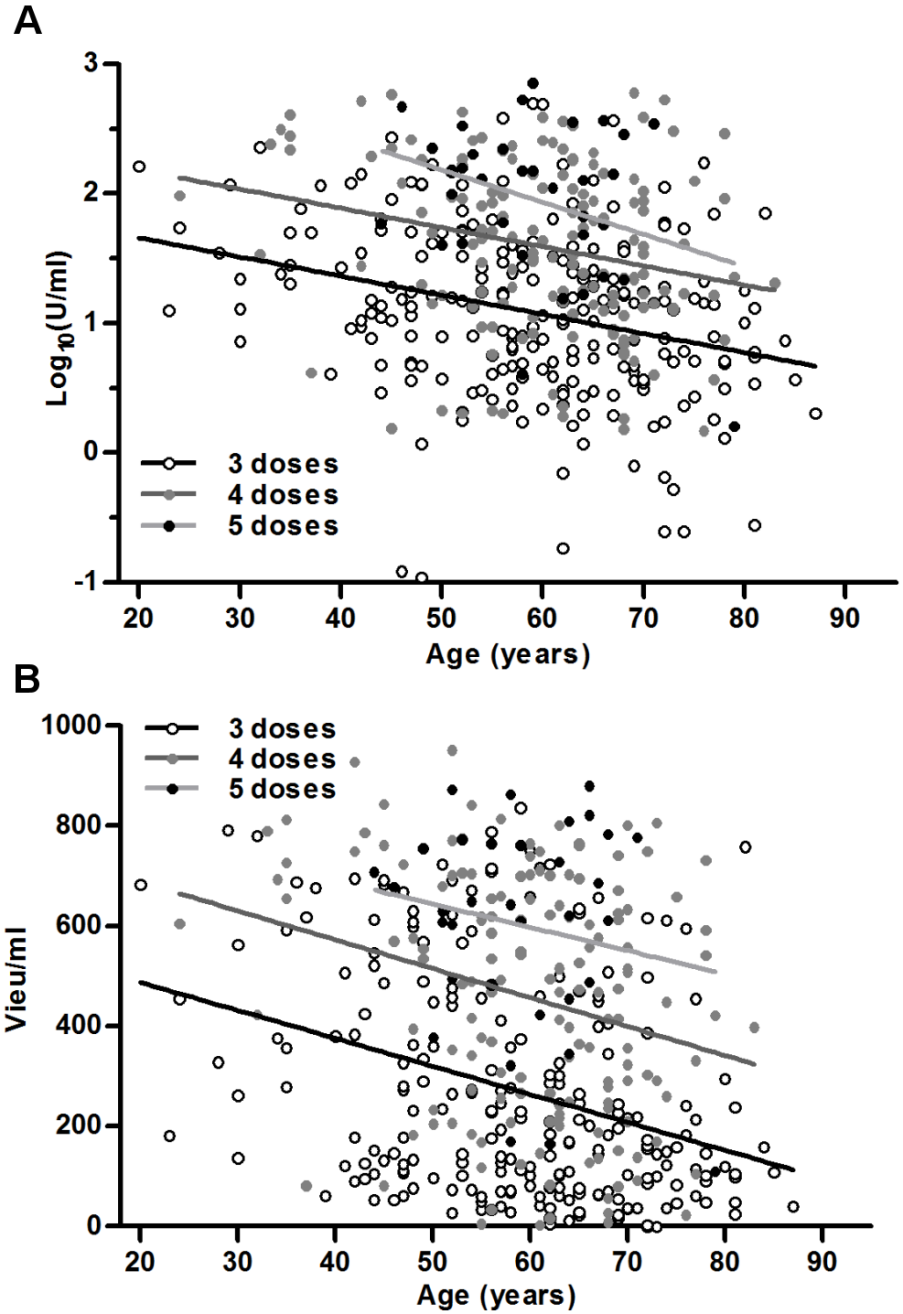
Linear regression of age versus antibody titer. Grouped by study participants that have received 3 (n = 227), 4 (n = 155) or 5 (n = 34) vaccine doses. Analyzed using: (A) Enzygnost, and (B) Immunozym.

For the study participants that had received 3 vaccine doses (n = 227), both the SPR (p<0.001, all 3 assays) (Figure 3: A), and the GMT (Enzygnost p = 0.001, Immunozym p<0.001) (Figure 3: C, E), decreased significantly with increased age. For the study participants that had received 4 vaccine doses (n = 155), the SPR did not decline significantly with age, i.e. most individuals even in the older age groups had a titer above the cutoff for seropositivity after 4 doses (Figure 3: B), and the GMT decline with increased age did not reach statistical significance for the subset of individuals that had received 4 doses (Figure 3: D, F). However, linear regression analysis without subdivision into age groups showed that the actual decline rate with regard to age was equal for those that had received 3 and 4 vaccine doses, −0.97 U/ml/year (p = 0.013) as measured using Enzygnost, and −5.6 VIEU/ml/year (p = 0.002) as measured using Immunozym (Figure 4: A, B). Binary logistic regression analysis showed that for each year of increased age, the mean odds of being seronegative as measured by all 3 assays increased by 1.03 (95% CI 1.01–1.06, p<0.001), for all the vaccinated participants.

### Gender and time since last vaccine dose

There was no difference in the antibody titer decline rate or overall antibody titer between women and men with regard to age. Also, there was no significant decline in antibody titer with regard to the time since last vaccination (0–4 years) for those that had received 3 or 4 doses.

### Self-reported health-related factors

Using binary logistic regression analysis we controlled for the other variables as possible confounding factors, most importantly age and number of vaccine doses received, and found that: Participants that were vaccinated against yellow fever or Japanese encephalitis had decreased odds of being seropositive for TBEV antibodies in the neutralizing antibody assay (RFFIT) 0.37 (95% CI 0.20–0.68, p<0.002), but not with the two ELISA assays that measures TBEV specific IgG antibodies.

Furthermore, participants with self-reported asthma had increased odds of being seropositive for TBEV antibodies in the neutralizing antibody assay (RFFIT) 3.32 (95% CI 1.21–9.07, p<0.020), but not with the two ELISA assays.

In addition, self-reported diabetes had a positive correlation to previous TBE disease, but only for women (p = 0.023). In total 3.6% of the women had diabetes (95% CI 1.8–6.3), but 2 of 7 or 29% (95% CI 3.7–71) of the women who previously had TBE also had diabetes. Diabetes was also positively correlated to previous vaccination against yellow fever or Japanese encephalitis, but only for men (p = 0.043). In total 4.9% of the men had diabetes (95% CI 2.1–9.4). But for men who had been vaccinated against yellow fever or Japanese encephalitis, 4 of 31 or 12.9% (95% CI 3.6–30) also had diabetes. These statistical significances are based on too few individuals to draw any conclusions regarding exposure to flaviviruses and diabetes. Correlations between all health-related factors were investigated but they were not significant.

### Bite by a TBEV infected tick

Three of the 533 participants in this study were bitten by ticks infected with TBEV as previously described [33]. The 3 ticks had been feeding for <12 h, <12 h, and 24–36 h, and the virus concentrations in the ticks were <400, <400, and 4,200 TBEV copies/tick respectively (Table 2). All 3 participants had been vaccinated with 3 doses before they were bitten by the positive tick. The measured IgG antibody titers within a few days after the tick was removed were higher in all individuals, than in their serum samples taken 3 months after the tick-bite. All were positive in the RFFIT neutralizing antibody assay at both time points. None of them developed any symptoms of TBE-infection.

**Table 2:** Analysis of 1^st^ (0-month) and 2^nd^ (3-month) serum from 3 study participants (each vaccinated with 3 doses), bitten by TBEV containing ticks.

Tick containing TBEV			Individual bittenby TBEV infected tick			Serum analysis results^b^					
						1^st^ serum			2^nd^ serum		
No.^a^	Feeding-time, h	TBEV copies	Age	Sex	Years sincelast vacc.	Enz	Imm	NT	Enz	Imm	NT
2	24–36	4200	57	F	1.8	24	350	+	22	260	+
4	<12	<400	49	F	1.7	30	440	+	29	380	+
5	<12	<400	35	M	3.9	35	580	+	14	330	+

a The tick number corresponds to the number in Table 3 of Lindblom et al. 2014 [33].

b Enz: Enzygnost (U/ml), Imm: Immunozym (VIEU/ml), NT: RFFIT (+/−).

### Evaluation of antibody assays

**Figure 5:**
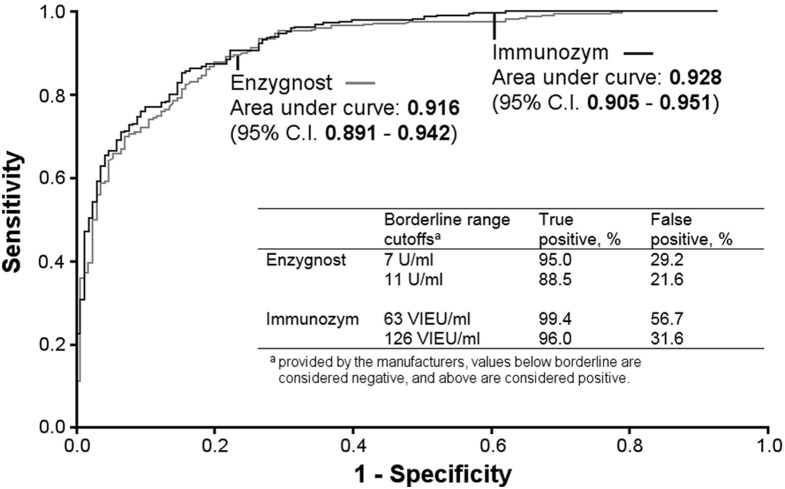
Receiver-Operating Characteristic (ROC) curves. Displaying the tradeoff between the sensitivity (true positive rate) and the 1-specificity (false positive rate), for Enzygnost and Immunozym for different cutoffs, in reference to the neutralizing antibody assay RFFIT as golden standard.

Of all 533 serum samples analyzed, the 3 assays (Enzygnost, Immunozym, and RFFIT) were in agreement in 81% of the cases, when borderline values in the two ELISA assays were regarded as negative. There was a significant difference in SPR between the Enzygnost and the Immunozym assay (p = 0.003), and the Immunozym and RFFIT assay (p = 0.004), but not between the Enzygnost and RFFIT assay. The Spearman correlation of all measurements of IgG antibody titers with the Immunozym and Enzygnost ELISA assay was 0.90 (p<0.001). A cross-comparison of the results between the two ELISA assays, including borderline values (Table 3), showed that 20 samples (3.8%) that tested positive in Immunozym tested negative in Enzygnost, 58 samples (10.9%) that were borderline in Immunozym were negative in Enzygnost, and 30 samples (5.6%) positive with Immunozym were borderline with Enzygnost. With borderline values regarded as negative in both ELISA assays the Immunozym assay indicated a positive test result for roughly 13% more samples than both the Enzygnost and the RFFIT assay. By using the RFFIT neutralizing antibody test as reference (golden standard), we calculated the sensitivity and specificity of the Enzygnost and Immunozym assay using ROC curve analysis (Figure 5). The overall accuracy, as measured by area under the curve (AUC), was not significantly different for the two assays [Enzygnost AUC = 0.92 (95% CI 0.89–0.94); Immunozym AUC = 0.93 (95% CI 0.91–0.95)]. However, the cutoff levels provided by the manufacturer for the Immunozym assay give a very high sensitivity, with a lower specificity as trade off (Figure 5), compared to the Enzygnost assay where the cutoff is chosen by the manufacturer to give a lower sensitivity resulting in a gain in specificity.

**Table 3:** Comparison of antibody analyses results between Enzygnost and Immunozym using the cutoffs defined by each manufacturer.

			Enzygnost		
		Negative	Borderline	Positive	Total
	Negative	65	1	0	66
**Immunozym**	Borderline	58	8	4	70
	Positive	20	30	347	397
	Total	143	39	351	533

## Discussion

The main findings of this study are that age and number of vaccine doses are the most important factors determining the immunological response to vaccination. According to our data, the antibody response to immunization declines linearly during life, which is in accordance with a previous study [17]. To compensate for the declining antibody response to each single dose of vaccine, older individuals needs to take additional vaccine doses in order to reach the same antibody titers as younger individuals. The average antibody titer reached by our older participants after 4 doses corresponds to the antibody titer reached after 3 doses by those 35 year’s younger. This reduced immune response with increased age is likely even larger, since the Åland Islands are highly TBE endemic and the background exposure to natural TBEV infection increases with age, which makes it more likely that the older participants have had a subclinical infection adding to their overall antibody titers. The background seroprevalence in the population is reported to be <5% in individuals under 20 years and increases to about 20% over a life-time [47]. The immune system, both the innate and the adaptive, deteriorates gradually with increasing age, which increases the susceptibility to infections and lowers the efficacy of immunization [48]. The B cell and T cell capacity to form long-term memory is impaired with increasing age [49], [50]. This could explain why TBE disease is generally more severe [1], [51], and vaccination failures are more frequent in older individuals [15], [25], [26]. It is noteworthy that the actual incidence rate of TBE was not reduced in those ≥50 years in Austria 10–20 years into the mass vaccination campaign that was started in 1981, while the number of TBE cases in those <50 years old was dramatically reduced [15]. To counteract this, an age adapted primary immunization program is needed, with sufficient number of vaccine doses, together with information regarding the increased risks for older individuals.

Of the self-reported factors, participants previously vaccinated against yellow fever or Japanese encephalitis had reduced odds of being seropositive for neutralizing TBEV antibodies, but not overall TBEV specific IgG antibodies. The reason for this is unclear, and also what significance this might have. In the WHO position paper on TBE published 2011 [11], it was specifically stated that more information is needed regarding the immune response in individuals who previously have been vaccinated against yellow fever or Japanese encephalitis. Participants with self-reported asthma had higher odds of being seropositive for neutralizing TBEV antibodies, but not overall for TBEV specific IgG antibodies. Asthma is a chronic inflammatory disease of the airways involving immune hypersensitivity [52]. However, we do not know how this could affect neutralizing antibodies against TBEV.

For the 3 vaccinated individuals that were bitten by ticks containing TBEV, the only noticeable effect was that the antibody titers were slightly higher in the serum sample taken a few days after the tick-bite, compared to the serum sample taken 3 months later. The explanation would perhaps be either a specific up regulation of TBEV antibodies due to immunological contact with the virus, or a general up regulation of the immune system in response to the tick-bite.

Since all our study participants from the Åland Islands had been vaccinated with the FSME-IMMUN vaccine, we could not investigate if there was any difference in sensitivity and specificity with regard to the Enzygnost and Immunozym assay as an effect of homologous or heterogeneous TBEV strains in the vaccine and ELISA assays, as reported by Jílková et al. [28]. The discrepancy between the Immunozym assay compared to the Enzygnost and RFFIT assay is fully accounted for by the difference in cut-off levels chosen as shown by the ROC curve analysis. It cannot be ruled out that the cutoffs for the Immunozym assay would yield another balance between sensitivity and specificity for individuals vaccinated with the Encepur vaccine, which we could not test. A high sensitivity with reduced specificity, as observed with the cut-off levels chosen by the manufacturer of the Immunozym assay, gives a high negative predictive value and is the ideal property for a rule-out test.

Fifteen participants had decided not to get vaccinated because they declared known TBEV seropositivity. These 15 participants where all seropositive and had high antibody titers, most likely as a result of mild or subclinical infection of TBEV.

The participants in this study volunteered on the basis of receiving a tick-bite, knowing about the study, and willingness to devote the time and effort. This resulted in an overrepresentation of women and older individuals compared to the general population on the Åland Islands. However, this selection bias gave us the opportunity to investigate TBEV vaccination in older individuals. We believe that the results obtained in this study have external validity regarding the declining immune response to immunization against TBEV in older individuals.

## Conclusion

The age of the vaccinated individual and the number of vaccine doses were the two most important factors determining the immunological response to vaccination. The proportion of seropositive individuals were significantly increased after 4 and 5 vaccine doses compared to 3 doses. The antibody response to TBEV vaccination declines linearly with increased age. Older individuals need 4 doses to reach the antibody titer a 35 year younger individual reaches after 3 doses, i.e. a 60 year old individual needs 4 doses on average to reach the same antibody titer that a 25 year old does with 3 doses. This should be considered when establishing vaccination guidelines.

## Supporting Information

Appendix S1 **Questionnaires answered by the participants at time of enrollment and the 3-month follow-up.**
(PDF)

Database S1 **Complete database of all included variables.**
(CSV)
