# Supplementary material for: Factors Determining Immunological Response to Vaccination against Tick-Borne Encephalitis Virus in Older Individuals
Source: PLoS One. 2014 Jun 26;9(6):e100860. doi: 10.1371/journal.pone.0100860 (PMC4072701; doi:10.1371/journal.pone.0100860)
Supplement: Appendix S1 — Questionnaires answered by the participants at time of enrollment and the 3-month follow-up. (PDF) [file pone.0100860.s001.pdf]

STING-study Code ……………………… Date ……………………

Questionnaire for you who have been tick-bitten

Please answer all questions!

When did you notice you had been tick-bitten?

Year-Month-Day: _________ _____ _____ Time _________

When do you think you were tick-bitten?

Year-Month-Day: _________ _____ _____ Time _________

In what town/city were you when you were tick-bitten?

____

What kind of nature type had you visited?

Lake/Sea ☐ Forrest ☐ Garden ☐ Lawn ☐

Other: ____

When was the tick removed?

Year-Month-Day: _________ _____ _____ Time _________

Where on the body was the tick attached?

____

Did you remove the whole tick? Yes ☐ No ☐ Don’t know ☐

Have you had any other tick bites this season? Yes ☐ No ☐ Don’t know ☐

If Yes, how many? 1-4 ☐ 5-9 ☐ >10 ☐

STING-study Code .... Date ....

Have you ever been treated for the tick-borne infection Borrelia?

Yes ☐ No ☐ Don't know ☐ If Yes; Year–Month–Day _________ _____ _____

Did you receive any medicine?

Yes ☐ No ☐ Don't know ☐ If Yes; which kind?___________________________

Have you ever been treated for the tick-borne infection Erythema migrans?
(Erythema migrans = red ring-like or homogenous expanding rash.)

Yes ☐ No ☐ Don't know ☐ If Yes; Year–Month–Day _________ _____ _____

Did you receive any medicine?

Yes ☐ No ☐ Don't know ☐ If Yes; which kind?__________________________

Have you ever been treated for the tick-borne infection Ehrlichiosis?
(Ehrlichiosis = flu-like symptoms)

Yes ☐ No ☐ Don't know ☐ If Yes; Year–Month–Day _________ _____ _____

Did you receive any medicine?

Yes ☐ No ☐ Don't know ☐ If Yes; which kind?_____________________________

STING-study Code .... Date ....

Have you ever been treated for the tick-borne infection TBE?
(TBE = a viral infectious disease involving the central nervous system.)

Yes ☐ No ☐ Don't know ☐ If Yes; Year–Month–Day________ ____ ____

Did you receive any medicine?

Yes ☐ No ☐ Don't know ☐ If Yes; which kind?________________________

Do you have any of the following diseases?

Asthma Yes ☐ No ☐ Don't know ☐

Allergy Yes ☐ No ☐ Don't know ☐

Diabetes Yes ☐ No ☐ Don't know ☐

Tumour-related disease Yes ☐ No ☐ Don't know ☐

Are you on medication? Yes ☐ No ☐
If Yes; which kind?

____

Do you smoke? Yes ☐ No ☐ Stopped smoking ☐ Year ________

If Yes, how many cigarettes per week? ________

How many years have you smoked? ________

Do you have any pets? Yes ☐ No ☐

Dog Yes ☐ No ☐

Cat Yes ☐ No ☐

Bunny Yes ☐ No ☐

Other: ____

Are you vaccinated for TBE? Yes ☐ No ☐ Don’t know ☐

If Yes; Year-Month-Day_________ _____ _____

Are you vaccinated for Yellow fever? Yes ☐ No ☐ Don’t know ☐

If Yes; Year-Month-Day_________ _____ _____

Are you vaccinated for Japanese encephalitis? Yes ☐ No ☐ Don’t know ☐

If Yes; Year-Month-Day_________ _____ _____

Thank you for your answers!

STING-study Code ……………………… Date ……………………

Hi!

You are a participant in our study ”Tick-bites and risk of disease”. We previously received blood samples from you and a filled in questionnaire. Now, three month later we need a follow up blood sample. The follow up blood samples can be taken at ____________________, week ___, Monday, Tuesday, Wednesday, or Thursday, between _____ and _____ a clock.

If you had additional tick-bites since the first one and you have collected the ticks in the tube with a yellow cork, then please take that tube with you to the blood sampling.

We would like to know if you have had any symptoms related to tick-borne diseases during the study period. Please answer the following three questions and write your name, birth date and telephone number on the next page. We might give you a telephone call if you reported symptoms. Take this paper and the test tubes to your primary health care center when you go for the sample-taking.

1) Have you had additional tick-bites since the first sample-taking?

Yes ☐ No ☐ Don’t know ☐

If yes; when? Year-Month-Day: ________ ____ ____

2) How have you been feeling in general since the first sample-taking?
Have you been feeling good/as usual?

Yes ☐ No ☐ Don’t know ☐

If no; please report if you had any of the following symptoms:

Headache	Yes ☐	No ☐
Fatigue	Yes ☐	No ☐
Fever, 38° or higher	Yes ☐	No ☐
Neck pain	Yes ☐	No ☐
Loss of appetite	Yes ☐	No ☐
Nausea/Vomiting	Yes ☐	No ☐
Weight Loss	Yes ☐	No ☐
Vertigo	Yes ☐	No ☐
Concentration difficulties	Yes ☐	No ☐
Radiating pain	Yes ☐	No ☐
Muscle or joint pain	Yes ☐	No ☐
Numbness/formication	Yes ☐	No ☐

Please turn the page!

STING-study Code .... Date ....

3) If you reported any symptoms in question 2, did the symptoms arrive before or after any additional tick-bites?

Before additional tick-bite Yes ☐ No ☐ Don't know ☐

After additional tick-bite Yes ☐ No ☐ Don't know ☐

4) If you reported any symptoms in question 2, did you visit you primary health care center for this?

Yes ☐ No ☐

5) If you reported any symptoms in question 2, how many days did the symptoms last?

____

Thank you for your answers!

Please make sure you answered every question!
Bring this paper to your new sample-taking!

Name: ____

Birth: ____

Telephone number:
Home ____
Work ____
Mobile ____

Best regards
xxx xxxx
